# Status of serine tyrosine kinase at germline and expressional levels in asthma patients

**DOI:** 10.22099/mbrc.2019.33040.1394

**Published:** 2019-06

**Authors:** Namoodn Sahar, Shakila Bibi, Nosheen Masood, Rani Faryal

**Affiliations:** 1 Department of Biosciences, COMSATS Institute of Information Technology, Islamabad, Pakistan; 2 Department of Environmental Sciences, Fatima Jinnah Women University, Rawalpindi, Pakistan; 3 Department of Microbiology, Faculty of Biological Sciences, Quaid-i-Azam University, Islamabad, Pakistan

**Keywords:** Spleen tyrosine kinase, Expression, SSCP, ELISA

## Abstract

Asthma is a disease marked by inflammation of airways with an increasing incidence rate worldwide especially among Asian population. Spleen tyrosine kinase (Syk) is known to be involved in regulation of such inflammation response and thereby rendering its inevitable importance among asthma patients. DNA extraction followed by PCR and sequencing was performed for genomic analysis, mRNA analysis was done by RT PCR whereas Western blot and ELISA was used for protein study. Image J and UNAFOLD were also used for Bioinformatics analysis.The mean age of patients and controls were 31.1±9.3 and 30.4±6.1 years respectively. Results of sequencing showed nonsense exonic mutations in exon 3 at g.25710G>A and g.25722G**>**A positions. Substitution mutations in introns were also found at g.25827G>A (intron 3), g.63425C>T (intron 8) and g.63445T>G (intron 8). Significantly increased levels of IgE and significantly decreased expression of Syk at transcriptional level was found in patients compared to controls. The western blot results of asthmatic samples and healthy controls revealed that Syk has comparatively low expression in diseased individual’s PBMCs. *SYK* has been found to be altered in DNA, mRNA and protein expression in asthma patients among Pakistani population therefore patients should be treated according to their Syk status for more effective recovery.

## INTRODUCTION

Asthma being a chronic inflammatory syndrome has many clinical phenotypes in both children and adults. It is affecting about 300 million people worldwide, a total that is expected to rise by an additional 100 million, mainly in children, over the next 15-20 years [1]. Asthma is mostly an allergic condition of the lungs [2]. Although the most important determinant for the development of allergy and asthma is a genetic predisposition but environmental factors such as exposure to allergens, infections, and air pollution, also play an important role in the development of allergic and asthmatic inflammatory responses [3]. 

Moreover, different cellular and molecular pathways are involved in the pathogenesis of asthma. Such pathways include those which are part of adaptive and innate immunity. Mast cells, TH2 cells, basophils, neutrophils, eosinophil are important cells in pathogenesis of allergic asthma [4]. IgE has emerged as a fundamental player in allergic responses and captured interest due to the evasive epidemic of allergies and asthma [5]. It has been discovered that IgE acts as part of a protein network, which includes its two primary receptors, FcεRI and FcεRII (CD23) [6]. Triggering of inflammatory cells plays an important role in the elicitation of the immediate phase of an allergic response leading to acute local responses such as edema formation, tissue swelling, or bronchoconstriction. By the release of chemotactic and pro-inflammatory mediators, mononuclear cells can also have an effect on the late phase responses of an allergic response [7]. 

Protein tyrosine kinases (PTKs) are an important family of enzymes that have the potential to effectuate a plethora of signaling cascades in various immune and non-immune cells, owing to their ability for tyrosine phosphorylation [8]. Spleen tyrosine kinase (Syk) is an important member of non-receptor PTKs, and associates with antigen receptor on inner side of plasma membrane [9]. A common structural feature of the Syk family is the presence of tandem N-terminal SH2 domains and a C-terminal catalytic region with important interdomain regions, A and B between these domains. The human Syk is a protein of 635 amino acids, in which the N-terminal SH2 domain spans amino acids 10-102, the C-terminal SH2 domain spans amino acids 163-254, and the kinase domain includes amino acids 366-621 [10]. The activation of tyrosine kinases is an early and essential event in the transduction of signals from immune receptors of myeloid lineage cells which contain ITAMs in their cytoplasmic domains. In mast cells, Syk interacts with FcεRI and becomes activated when FcεRI is cross-linked [11]. 

The phosphorylated ITAM mediates association of the receptor complex with signaling molecules containing SH2 domains triggering the activation of various nodes of inflammation [12, 13]. Syk S, a splice variant of *SYK* is found both in normal and in tumor cells [14]. Syk S has similar kinase activity as Syk to phosphorylate some substrates such as Cbl and Plcγ1 but it is unable to couple the stimulation of FcεRI on basophils or antigen receptor on T cells to cellular activation because of its inability to phosphorylate downstream targets, due to a 23 amino acid deletion in interdomain B [15]. The prevalence of allergic diseases, including asthma, is quite high in Pakistan [16]. 

The aim of the present study is to identify the polymorphisms that may be associated with the Pakistani population and the expression patterns involved in the manifestation of allergic asthma in Pakistani population. As there is no availability of data related to Syk expression in asthmatic patients of Pakistan, there is a need to assess role of Syk among asthmatic population.

## MATERIALS AND METHODS

**Sampling and inclusion/exclusion criteria:** Asthmatic blood samples of 270 patients were collected from National Institute of Health (NIH), Islamabad, Pakistan. An approval from ethical committees of NIH and COMSATS Institute Information Technology, Islamabad, Pakistan was taken prior to start of study. The individuals diagnosed of asthma and positive skin prick tests were selected as patients of allergic asthma. The individuals with no symptoms or history of atopy or asthma and were negative for skin prick test were taken as controls (n=200). For blood sampling, a consent form was signed by the patients and controls.

**Genetic analysis:** DNA from whole blood was isolated by proteinase K incubation and phenol-chloroform extraction [17]. Following DNA isolation, PCR was performed on all patients and controls using primers designed by Primer3 software for *SYK* gene. The PCR products were analyzed for Single Strand Conformation Polymorphism (SSCP) on PAGE. SSCP results were analyzed using gel documentation system (BioDocAnalyze Biometra) after ethidium bromide staining. Variants were selected based on mobility shift and banding pattern. These variants were sent for sequencing to MC LAB San Francisco, USA.

**mRNA analysis:** For expressional analysis of mRNA, peripheral blood mononuclear cells (PBMCs) were extracted from the fresh samples of asthmatic and healthy individuals with gradient centrifugation using Histopaque. RNA from these cells was extracted using TRIzol. Superscript III First-Strand Synthesis System for RT-PCR was used for cDNA synthesis from DNase treated RNA. *SYK* gene was amplified to analyze its expression using Forward Primer (5’-3’) CCA CTG TGG CCA GCA CGA GG and Reverse Primer (3’-5’) GAT GCC ACC AGG GCA GCC TG. Actin was amplified as an internal control. 

**Protein analysis:** Serum was isolated from blood in non EDTA vacutainers and centrifuged for 10 minutes. Serum was collected in a separate tube. Total IgE levels from serum of asthma patients and healthy controls were determined by using MicroLISA^TM^- IgE kit (Amgenix). 

For protein analysis, ProteoJET^TM^ Cytoplasmic and Nuclear Protein Extraction Kit were used to extract proteins from PBMCs of asthmatic and healthy individuals. Protease inhibitors were added to prevent any proteolysis. The extracted protein lysates were subjected to protein estimation using Bradford Reagent. The spectrophotometer was used to detect the absorbance of the protein at 595 nm. For western blotting, the proteins were resolved on 10% SDS-PAGE and immediately transferred to nitrocellulose membrane using Towbin/transfer buffer [18]. Using Ponceau stain the membrane bound protein bands were visualized. For immunoblotting Syk 4D10 (1:2000 dilution), as primary antibody and HRP conjugated mouse IgG (1:10,000 dilution) as secondary antibody were used.

**Bioinformatics analysis:** Image J software for image processing was used to verify the band intensities as a result of semi-quantitative RT-PCR and western blots [19]. The software generated peaks according to the band intensities and the expression was compared between patient samples and controls. UNAFOLD (Integrated DNA technology server) was used to predict the structure and stability of secondary structure of *SYK* pre-mRNA to assess the effect of mutations found in exon-4 encoding 5́’ UTR region. Best structure was selected on the basis of lowest ΔG value. To analyze the stability of original and mutated secondary structure of *SYK* pre-mRNA, the optimal temperature and sodium ion concentration was set at 50°C and 15mM, respectively to predict mRNA that remains intact *in vivo*.

**Statistical analysis** **:** All the data was analyzed using SPSS 21.0. Categorical variables were represented by mean and S.D. Descriptive statistics and chi square test was applied to find the significance and results with P<0.05 was considered as statistically significant. 

## RESULTS

The patients had mean age of 31.1±9.3 years and included 59% females. Whereas the healthy control individuals had mean age of 30.4±6.1 years with 60% females. 

**Figure 1:**
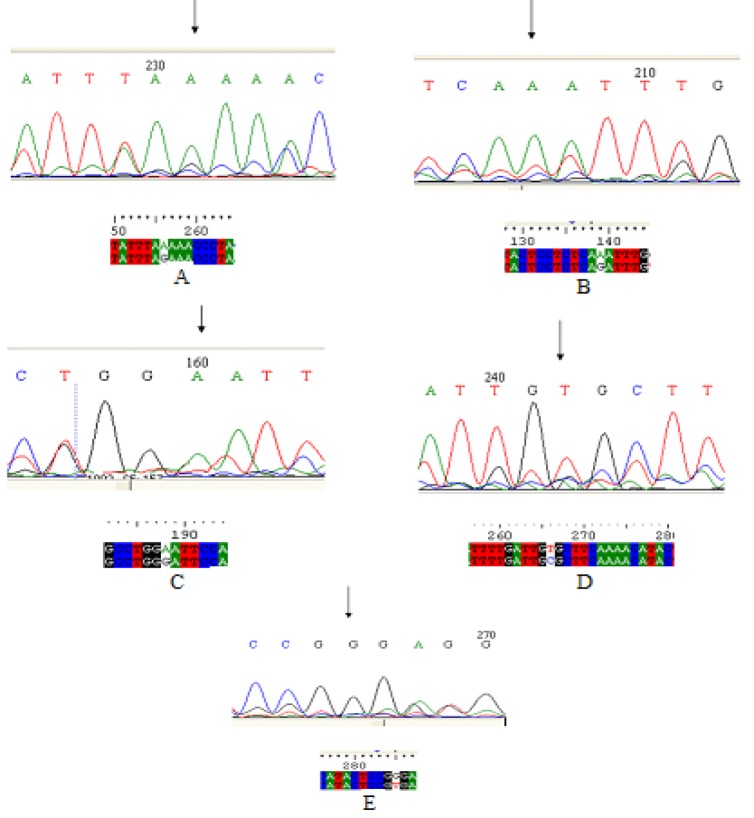
Mutations in SYK gene after SSCP and subsequent sequencing in allergic asthma patients showing A) substitution mutation in intronic region of exon 3 (25827 G>A); B) substitution mutation in exon 3 (25710 G>A); C) substitution mutation in exon 3 (25722 G>A); D) substitution mutation in exon 8 (63425 C>T) and E) substitution mutation in exon 8 (63445 T>G)

Total 66 samples showed variation in mobility pattern on SSCP gel. After sequencing of these samples five novel mutations were identified in asthma patients which were all substitution of single nucleotide. Two mutations were identified in exonic region of asthma samples. Three mutations were found in intronic region. Exonic mutations were found in exon 3 and they were at g.25710G>A and g.25722G>A positions (Fig. 1). Both of these exonic mutations were nonsense mutations resulting in same amino acid (valine in first case and proline in second case) as before mutation. In exon 3, g.25710G>A and g.25722G>A mutations were significantly related to these parameters as well. These mutations were significantly present in patients of age below 30 years who were females and non-smokers. Substitution mutations in intron were g.25827G>A, g.63425C>T and g.63445T>G; first was in intron 3 and other two in intron 8 (Fig. 1). 

Significant number (44, 40 and 60 respectively) of patients showed these mutations compared with controls. Mutation g.25827G>A in intron 3 region is significantly related to age, gender, smoking and family history. This mutation is present in patients of thirty and below thirty years of age (P<0.05). Significant results showed that g.25827G>A mutation is strongly associated with age factor. This mutation is also highly prevalent in females as compared to males in our population (P<0.05). In intron 8 two mutations were identified g.63425C>T and g.63445T>G. Mutation g.63445T>G was significantly associated with nonsmokers and number of allergen types. Mutation g.63425C>T was significantly associated with age and gender. This mutation was significantly present in patients both over and below thirty year of age, mostly being females.

**Figure 2:**
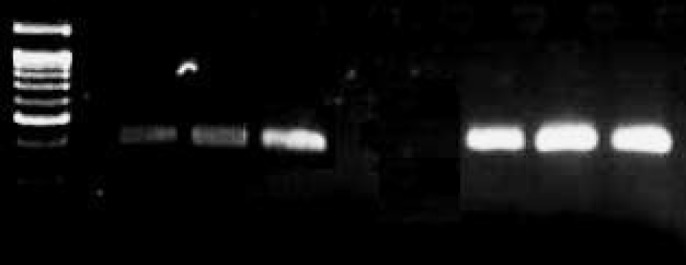
Semi-Quantitative RT-PCR with actin as housekeeping gene, 1^st^ lane has ladder followed by 3 patients samples and then ending 3 control samples showing expression of SYK mRNA

mRNA expression levels were compared between patients and controls. The bands on agarose gel showed decreased expression of *SYK* mRNA as compared with controls (Fig. 2) whereas housekeeping gene (actin) expression was similar between compared samples. The bands of actin and Syk amplified products were quantified using Image J software. This software produced high peaks for Syk in control samples as compared to asthmatic patient samples. Although peaks for actin were similar in all the samples. Results showed significantly decreased expression of *SYK* at transcriptional level in patients (P<0.05).

The results of ELISA were calculated as mean of log IgE with controls having 1.82±0.50 IU while patients had 2.08±0.66 IU concentration of IgE in the serum samples. Significantly increased levels of IgE were observed in patients compared to controls (P<0.05).

**Figure 3:**
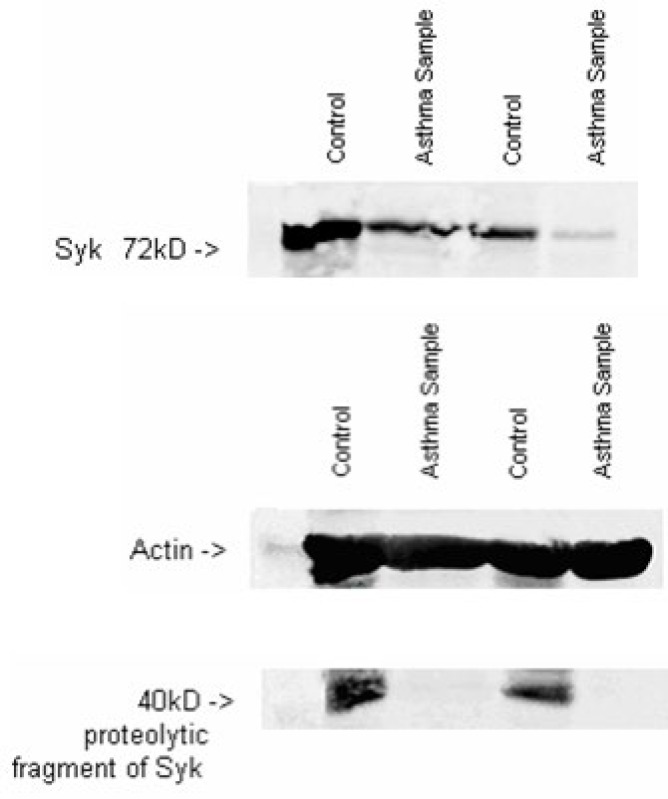
Immunoblot of whole cell lysate of PCBMs probed with anti-Syk4D10. Actin used as internal control

The western blot results of asthmatic samples and healthy controls revealed that Syk has comparatively low expression in diseased individual’s PBMCs. Moreover, in healthy PBMCs, along with 72kD Syk enzyme a proteolysis fragment of Syk was repeatedly seen in blots at 40kD, which was absent in asthmatic PBMCs (Fig. 3). It showed the presence of Syk S, 68kD isoform of enzyme in PBMCs lysates. These bands were detected when high amount of total proteins were loaded in each of the SDS-PAGE wells i.e. around 35-45 µg per well. These western blots were quantified using Image J software. This software produced high peaks for Syk in healthy individual sample as compared to asthmatic samples for which it generated low peaks.

Bioinformatics analysis was carried out to analyze the effect of mutation in pre-mRNA structure and stability. For this purpose, an UNAFOLD server was used to predict pre-mRNA structure of Syk. For exon 3, the pre-mRNA predicted structures of normal and mutated Syk had different stabilities. Two mutations were introduced in originals sequence of exon 3 of Syk pre-mRNA separately and collectively, and their structures were analyzed. One mutation g.25710G>A, didn't show any effect on structure and stability of pre-mRNA. Structure and stability were effected through induction of second mutation g.25722G>A. Mutated model showed different values of ΔG and Tm (-0.12 kcal.mol^-1^, 51.1 °C) as compared to original structure of Syk pre-mRNA with (-1.08 kcal.mol^-1 ^and 59.5^o^C). ΔG is the possibility of structure formation which was higher in mutated structure, which concluded that mutated structure is more stable. Through structural analysis it was observed that mutated model has longer stem with high GC content as compare to original value. On the basis of ΔG (possibility of forming a secondary structure), T_m_ and length of stem loop along with GC/AT content, the mutated structure was found to be more stable (Table 1; Figure S1). As stability of pre-mRNA increases, more energy will be required to linearize it, and binding of promoter binding protein with pre-mRNA will be affected.

**Table 1:** Comparison of physiochemical properties of secondary pre mRNA structure predicted by UNAFOLD of SYK 5́ UTR and mutated 5́ UTR after introduction of mutation at position 26, 414 G>A in exon 4

	**Original SYK**	**Mutated SYK**
ΔG	-1.08 kcal.mol^-1^	-0.12 kcal.mol^-1^
Tm	59.5 °C	Tm=51.1 °C
ΔH	-37.7 kcal.mol^-1^	-36.8 kcal.mol^-1^
ΔS	-113.32cal.k^-1^.mol^-1^	ΔS= -113.51cal.k^-1^.mol^-1^

## DISCUSSION

*SYK* is reported to be a tumor suppressor gene and its expressional abnormality is also associated with various allergies [20]. In respiratory allergies, *SYK* is known to be up-regulated and inhibitors have been used as therapies [21]. As these cells are mainly found on inflammatory sites, PBMCs were focus of this research to investigate their role in allergic asthma. The intracellular mechanisms to trigger asthma and allergy constitute a plethora of signaling cascades in different tissues and cells arising from transmembrane proteins acting as receptors. In turn, a number of receptor PTKs and non-receptor PTKs are recruited in different cells to accomplish the interplay of cytokines and inflammatory mediators for asthma and allergy. The abnormalities in cell signaling kinases make them the culprit for pathogenesis of various diseases including cancers and allergies [22, 23]. These significant findings compelled us to examine the mutations of *SYK* kinase gene in local population as well as change in expression of Syk at the transcriptional and translational level in asthmatic patients. Our study is in accordance with work of Schatz and Camargo [24], they reported that asthma is to be more common in females than males. There is considerable evidence that sex hormones play a role in inflammation, specifically in the genesis and course of asthma [25]. In addition it is well documented that during pregnancy the clinical manifestation of asthma can change [26]. 

Mutations have been found in the exonic and intronic regions of *SYK* gene in asthmatic patients. These mutations were present in exon 3 of *SYK* gene which is located in 5’ untranslated regions (5’UTRs) that contain genetic information for posttranscriptional regulation of gene expression. These regions have important functions in gene regulation and their mutations effecting structure and stability of pre-mRNA is a major factor of regulation and expression [27]. The results of present study are in accordance with previous studies [28, 29]. Mutations in introns cannot be ignored because they are not considered as junk DNA now and have been related in regulation of genes. Therefore, these mutations cannot be ignored in pathogenesis of asthma. In asthmatic patients, the presence of comparatively less intense bands of *SYK* amplified product were observed suggesting low presence of *SYK* in transcriptome. Whereas, on the other hand among healthy individuals more intense bands of *SYK* amplified product were present. A study in human breast cancer also revealed reduced expression of *SYK* gene to be associated with poor prognosis [30]. However, in this study this reduction may owe to different genetic mutations of *SYK* genes in local population.

The expression of Syk in 23 out of 25 asthmatic patients was lower as compared to Syk expression in healthy individuals, where expression of Syk was higher. Previous studies on non-releaser basophils of Syk showed that Syk mRNA levels were normal and treatment of these basophils with proteasome inhibitors restores Syk expression. These studies clearly depict that the lower Syk expression is primarily due to excess Syk degradation [31, 32]. But in our present study, the lower expression of Syk is consistent with the lower expression of RNA as well. In a recent work by Harvard *et al*., [33] in 2011, it was established that variability in Syk expression does not readily explain the variability in IgE-mediated histamine release which is a key factor in asthma inflammation. This further strengthens the role of population specific genetic mutations regulating expression. Contrary to our findings, there are studies which showed that Syk inhibitors were used for targeting Syk in asthma therapy, suggesting high expression in asthma [34, 35]. 

In a recent study, histamine non-releaser basophils from non-asthmatic individuals had excessive proteolysis of Syk, but the amount of intact Syk was also low in these samples [36]. The western blot analysis of our collected samples also revealed the presence of a proteolytic fragment of Syk at 40kD. This fragment was only detected in healthy individual’s protein samples while it was absent in asthmatic samples. The presence of this fragment depicts a well establish regulation of Syk in signaling cascades, which is needed to be well studied further. In addition to 72kD Syk L, there was another variant of Syk that is 68kD (Syk S) which is expressed in some tissues. It was detected when high amount of proteins per well was loaded. This clearly showed the presence of Syk S in PBMCs but at low level. Various studies have shown that Syk S was unable to trigger the stimulation of FcεRI on basophils or antigen receptor on T cells to cellular activation because of its inability to phosphorylate downstream targets [9]. So at this stage no significant conclusion can be drawn regarding the presence of Syk S in our samples, further work is needed to analyze its role among asthma patient of Pakistani origin. 

The DNA mutations include functional elements comprising of stem-loop structures, upstream initiation codons, open reading frames, internal ribosome entry sites and various cis-acting elements. So, any mutation in these regions might affects structure and stability of pre-mRNA which will be major determinant for gene regulation and expression which was depicted in the *in-silico* analysis showing more stable protein structure of exon 3 mutation. 

## Supplementary Materials

Supplement
