# Supplementary material for: Status of serine tyrosine kinase at germline and expressional levels in asthma patients
Source: Mol Biol Res Commun. 2019 Jun;8(2):69–77. doi: 10.22099/mbrc.2019.33040.1394 (PMC6715266; doi:10.22099/mbrc.2019.33040.1394)
Supplement: Supplement [file mbrc-8-069-s001.pdf]

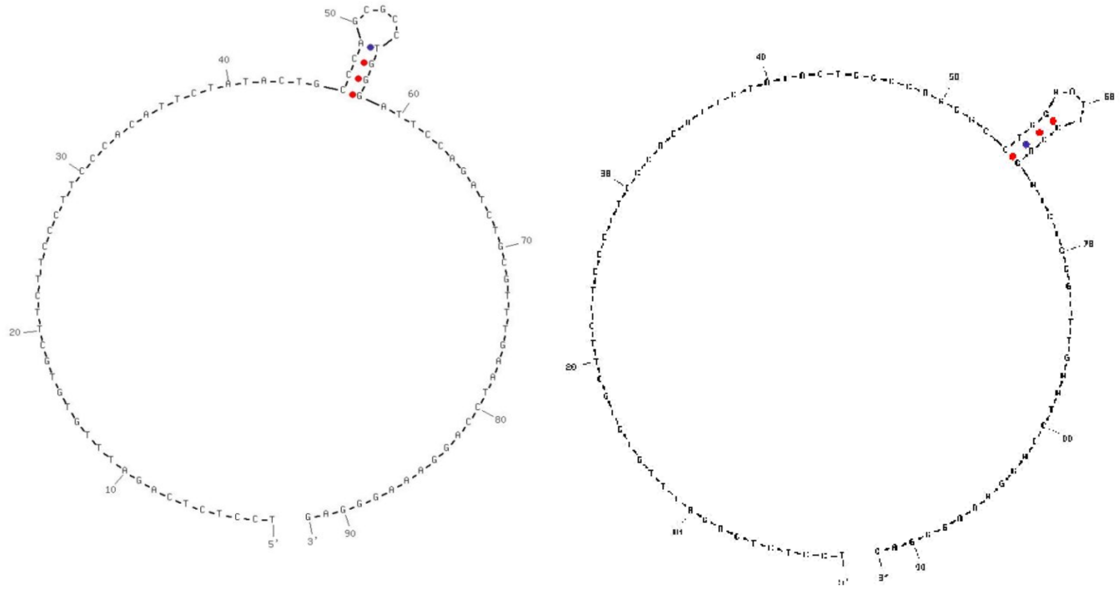

A **B**

Figure 1: Predicted secondary structure of pre-mRNA of exon 4 of SYK gene by using UNAFOLD tool. A) Stem was formed due to base pairing of three nucleotide starting from 46th nucleotide to 58th with a bulge of five nucleotides B) after introduction of mutation at position 26, 414 G>A structure and position of stem loop has changed.
